# Bibliometric analysis of high-intensity focused ultrasound

**DOI:** 10.3389/frma.2026.1849512

**Published:** 2026-07-27

**Authors:** Min Li, Rong Ou, Wanhui Zheng

**Affiliations:** 1 Department of Medical Education and Research, State Key Laboratory of Ultrasound in Medicine and Engineering, Beibei Affiliated Hospital of Chongqing Medical University, Chongqing, China; 2 Chongqing Medical University Library, State Key Laboratory of Ultrasound in Medicine and Engineering, Chongqing Medical University, Chongqing, China; 3 Hospital Cost Management Research Center, Beibei Affiliated Hospital of Chongqing Medical University, Chongqing, China

**Keywords:** bibliometric analysis, high-intensity focused ultrasound, knowledge mapping, research trends, visualization

## Abstract

**Purpose:**

This study analyzes global literature on High-Intensity Focused Ultrasound (HIFU), presenting its research distribution, thematic domains, and developmental trajectory.

**Methods:**

Data from the Web of Science were analyzed using InCites, CiteSpace, and VOSviewer to examine co-occurrence patterns, research hotspots, and citation bursts among countries, institutions, authors, and keywords.

**Results:**

HIFU research progressed slowly from 1994 to 2002, experienced rapid growth from 2003 to 2012, and entered a stable, high-output phase from 2013 to 2024. The United States and China led in both research output and impact, whereas Europe showed lower output but outstanding research quality. The primary research directions encompassed basic and clinical studies, technical optimization, and evaluations of safety and efficacy.

**Conclusion:**

Over the past three decades, HIFU research has evolved from foundational exploration to rapid expansion and technological maturation. Global HIFU research now presents substantial opportunities, driven by expanding clinical indications, growing demand for non-invasive therapies, and integration with imaging guidance, drug delivery, and precision medicine. However, its transition toward standardized clinical application still requires higher-quality evidence, large-scale multicenter studies, greater methodological rigor, and further improvements in treatment reproducibility, long-term efficacy, and international standardization.

## 1 Introduction

HIFU is one of the most important representative technologies in minimally invasive medicine. By delivering ultrasound energy externally and precisely focusing it on internal lesions, HIFU directly ablates pathological tissue through thermal or cavitation effects. This technique offers true non-invasiveness, high precision, and a favorable safety profile. In 1942, American scientist Frank Fry proposed the theoretical foundation for HIFU (Lynn et al., 1942; Wall et al., 1951; Fry et al., 1958). However, technological breakthroughs remained elusive. In the 1990s, Professor Zhibiao Wang of Chongqing Medical University developed, in China, the world's first HIFU system for tumor treatment; its successful use in treating patients with bone tumors marked a major breakthrough in HIFU technology (Wang, 2015, 2024). Subsequently, major global economies accelerated the development and application of HIFU, and related publications increased substantially. To objectively present the global distribution of HIFU research, this study analyzes HIFU-related publications using bibliometric methods.

## 2 Methods

### 2.1 Data sources

The Web of Science Core Collection database (WoS) indexes high-quality international scientific literature and provides not only numerous HIFU-related journal articles but also complete bibliographic and citation information for journals and articles (Falagas et al., 2008). For bibliometric analysis, the WoS database can automatically generate files in BibTeX format that can be readily recognized by bibliometric software. Therefore, WoS was selected as the data source for this study (Aria and Cuccurullo, 2017).

### 2.2 Data retrieval

**Figure 1:**
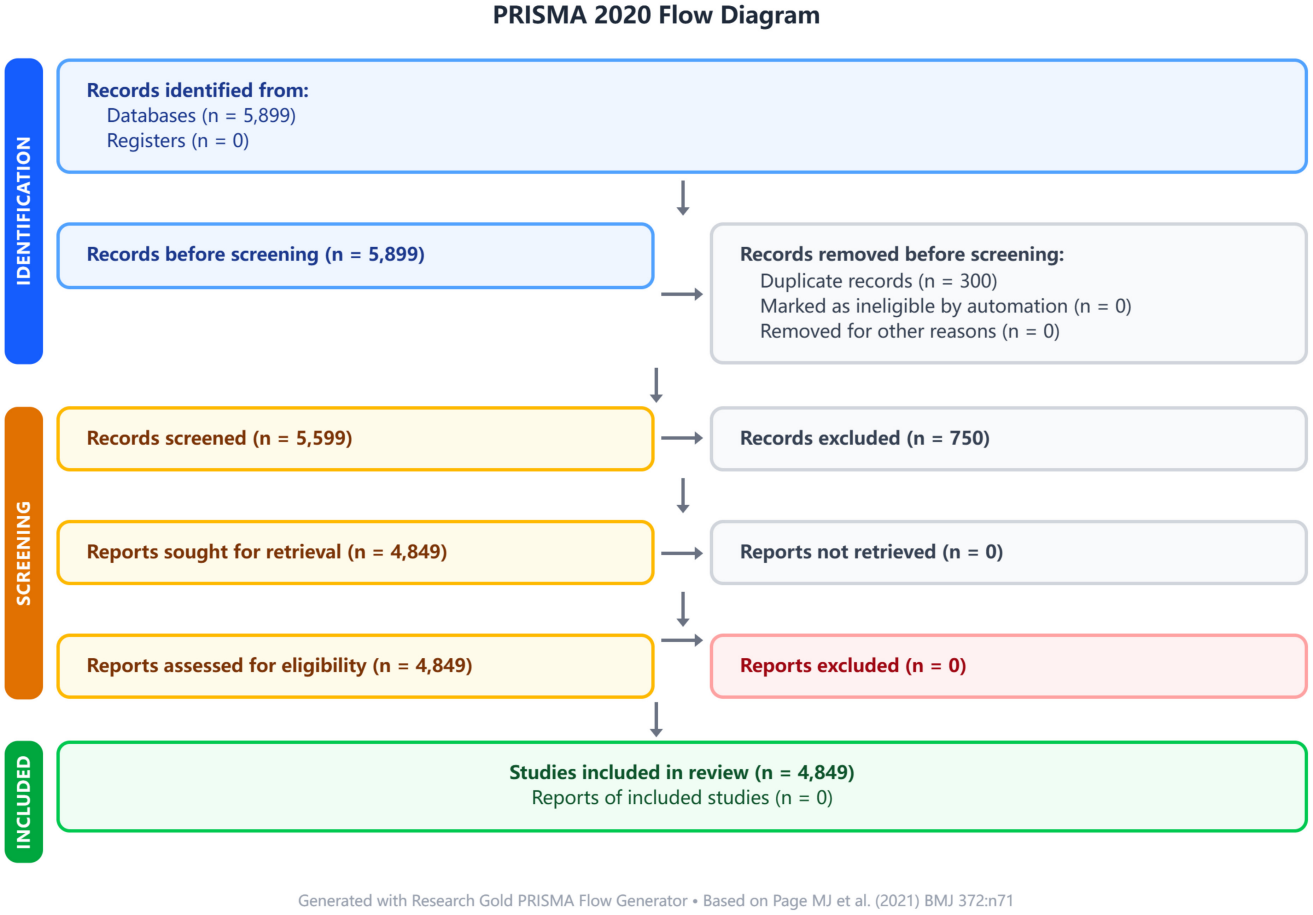
PRISMA 2020 flow diagram of the literature search and screening process. The initial database search identified 5,899 records, of which 300 duplicates were removed before screening. After title and abstract screening, 750 records were excluded, leaving 4,849 records for full-text assessment. All full texts were retrieved, and no additional records were excluded during the eligibility assessment. Finally, 4,849 records were included in the analysis.

As shown in Figure 1, First, HIFU-related subject terms were selected as search terms and then combined using the Boolean operators “AND” and “OR.” The combined search strategy was as follows: TS = (“HIFU” OR “high-intensity focused ultrasound”) AND PY = (1994–2024). The search was conducted on August 10, 2025, and covered publications from 1994 to 2024. Through this process, 5,899 records were obtained.

Second, the literature screening process was performed in accordance with the PRISMA 2020 statement. Initially, 5,899 records were retrieved from the Web of Science Core Collection. After 300 duplicates were removed, 5,599 unique records were screened based on their titles and abstracts. Among these records, 750 were excluded because they addressed irrelevant topics, were classified as non-research documents, or lacked key information. The remaining 4,849 records were assessed for eligibility through full-text review, and no further records were excluded at this stage. Finally, 4,849 studies were included in the bibliometric analysis.

Third, the filtered data were imported into bibliometric software for analysis. Finally, the analytical results were summarized, and the current shortcomings, challenges faced by HIFU, and future research directions were presented.

### 2.3 Research methods

This study used InCites, a research evaluation and analysis tool developed by Clarivate Analytics, to analyze research institutions, countries, and authors. The results were visualized using the bibliometric software CiteSpace 6.1.R6 and VOSviewer 1.6.19.

CiteSpace settings: We used CiteSpace 6.1.R6 for bibliometric analysis. The time span was set to 1994–2024, with 1-year time slices. The g-index was set to *k* = 20, and keywords, institutions, and authors were filtered using a threshold of at least 20 occurrences per slice (Chen, 2006).

VOSviewer settings: Co-authorship analysis was performed at the author and institution levels. For authors, the threshold was set at a minimum of 22 publications, and the top 55 authors were retained. For institutions, the threshold was set at a minimum of 26 publications, and the top 50 institutions were retained (van Eck and Waltman, 2010). These parameters were chosen to balance network clarity and the inclusion of core contributors, consistent with common practices in bibliometric studies.

### 2.4 Indicator definitions

The Category Normalized Citation Impact (CNCI) is a normalized metric that measures the citation impact of a paper or research entity; it accounts for differences in discipline, publication date, and document type. The CNCI is scaled such that 1 serves as the baseline. A CNCI greater than 1 indicates that the citation performance of a paper or research entity exceeds the global average, the higher the value, the greater the citation impact. A CNCI equal to 1 indicates that the citation performance is on par with the global average. A CNCI less than 1 indicates that the citation performance falls below the global average, suggesting relatively limited academic impact (Clarivate, 2025).

The Collaboration Category Normalized Citation Impact (Collab-CNCI) is an extended bibliometric indicator introduced by Clarivate's Institute for Scientific Information (ISI) in 2020. It builds on the standard CNCI by adding a fourth normalization layer for collaboration type, including domestic single-country collaboration, domestic multi-institutional collaboration, and international bilateral, trilateral, and quadrilateral-or-higher collaboration, eliminating the bias of varying collaboration patterns on citation performance. A Collab-CNCI value of 1 represents the global average citation impact for publications in the same subject category, publication year, document type, and collaboration type. Values greater than 1 indicate above-average citation impact within the same collaboration type, whereas values less than 1 indicate below-average citation impact.

This study uses patent citations as an indicator of technological influence. This metric counts the total number of times publications associated with a given entity are cited in patents, effectively measuring the technological impact, industry recognition, and commercialization value of research outcomes. It thereby addresses the limitations of evaluating research output solely based on the number of published papers (Manjunath et al., 2021). All relevant raw data were sourced from the InCites database, with the search period, strategy, and field-specific paper data kept consistent throughout the study. Regarding sample selection, at the national and institutional levels, the top ten entities worldwide in terms of HIFU-related paper output were selected as the research subjects. As these entities also rank among the top in the field in terms of patent citation counts, this approach ensures both sample representativeness and analytical consistency. However, at the author level, given the weak correlation between publication output and patent citation counts, as well as the asynchronous development patterns of these two metrics, this study did not adopt a ranking based on publication volume. Instead, patent citation counts were directly calculated for core authors in the field to objectively reflect differences in research orientation among scholars.

## 3 Results

### 3.1 Publication status

#### 3.1.1 Annual publication trends

**Figure 2:**
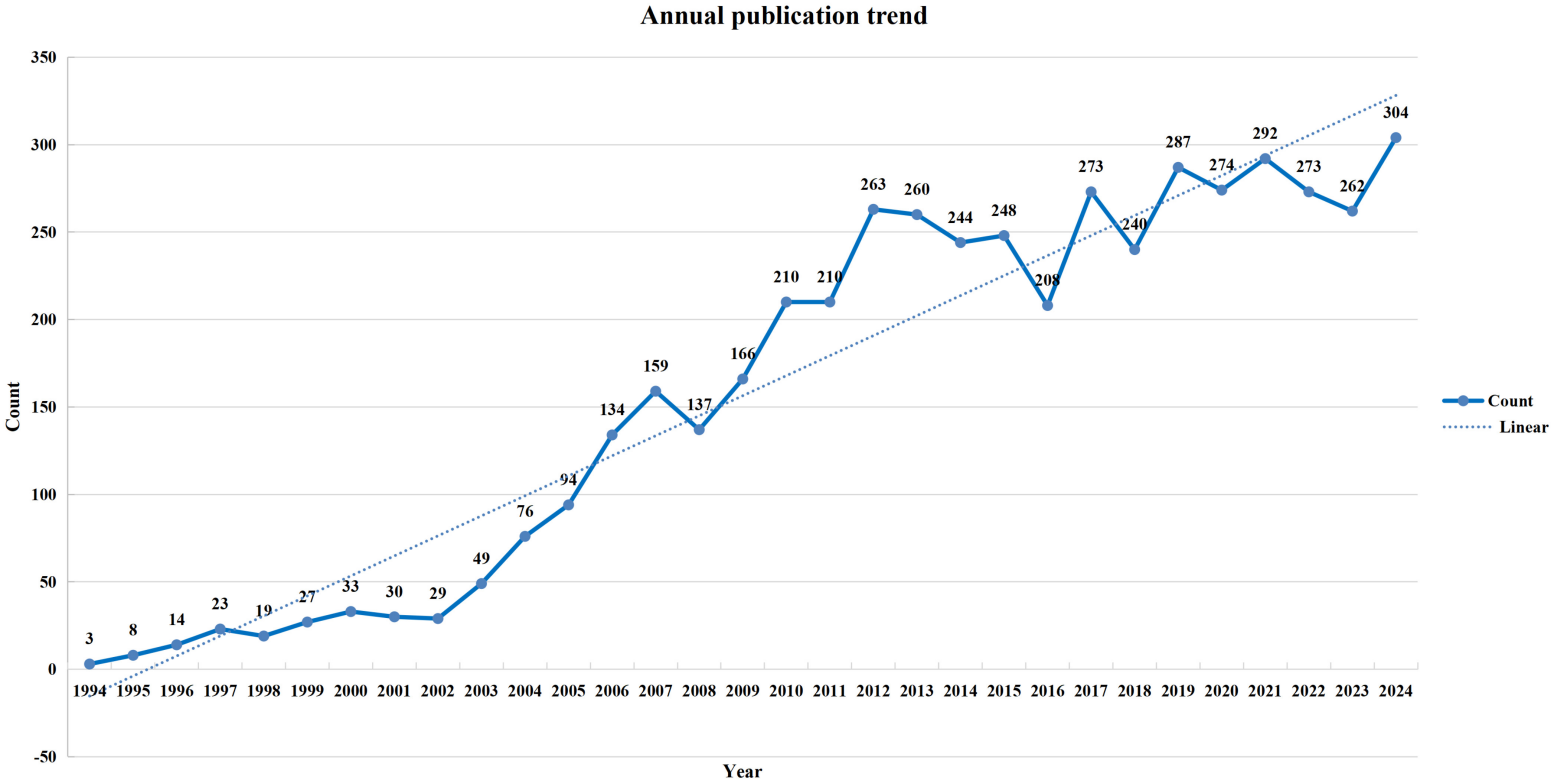
Annual publication trends in high-intensity focused ultrasound (HIFU) research from 1994 to 2024. The solid blue line represents the number of publications per year, whereas the dashed line indicates the overall linear growth trend. Three distinct phases can be observed: (1) a slow initial growth phase (1994–2002), during which annual publications remained below 30; (2) a rapid expansion phase (2003–2012), characterized by a steep increase from 49 to 263 publications per year; and (3) a sustained high-output phase (2013–2024), during which annual publications fluctuated between 208 and 304, maintaining a consistently high level of research activity.

Figure 2, the publication trend chart, illustrates the evolution of global research output in the field of HIFU from 1994 to 2024, clearly revealing the developmental trajectory and dynamic trends of this research domain. From 1994 to 2002, HIFU research output increased slowly, with an average of 21 publications per year. In 1994, only three papers were published. Although there was a slight increase in the following years, the annual number of publications reached only 29 in 2002. From 2003 onward, the publication volume showed a more pronounced upward trend. In 2003, 49 papers were published, and the annual publication count continued to increase, reaching 159 in 2007 and marking the first peak. In 2008, the number of publications declined slightly to 137, compared with 159 in 2007. However, from 2008 to 2012, the number of publications increased rapidly, reaching 263 articles in 2012 and forming a second peak. From 2013 to 2024, the annual publication volume remained generally high and rose steadily, despite some fluctuations, reaching 304 articles in 2024.

#### 3.1.2 Research countries

Based on regional publication data from InCites, this study analyzes key metrics in the HIFU field, including Web of Science publication volume, citation counts, and CNCI values, to examine the global distribution of HIFU research.

**Figure 3:**
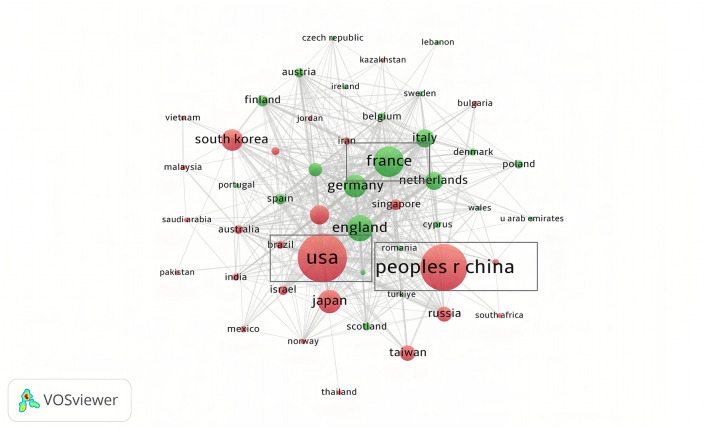
Co-authorship network of countries/regions in HIFU research, generated using VOSviewer. Each node represents a country/region, and node size is proportional to publication output. Links indicate collaborative relationships, with line thickness reflecting collaboration strength. Node colors represent distinct collaboration clusters rather than the average publication year. The United States and the People's Republic of China form the core of the network, while France, England, Germany, the Netherlands, Italy, Japan, and South Korea also occupy prominent positions, indicating extensive international collaboration.

As shown in Figure 3, larger nodes in the VOSviewer diagram indicate higher publication volumes, with the United States, China, and France representing the main contributing countries in HIFU research. As shown in Table 1, the United States ranks first with 1,325 publications, followed by mainland China with 1,150 and France with 504.

**Table 1:** Metrics including citation counts, CNCI values, and patent citations for the top ten regions by publication volume.

Rank	Name	Web of science documents	Times cited	Collab-CNCI	% Docs cited	CNCI	Citations from patents
1	USA	1325	36220	1.04	88.60	1.12	1731
2	CHINA MAINLAND	1150	21724	0.89	85.48	0.82	218
5	FRANCE	504	14517	0.96	86.90	1.10	405
3	UNITED KINGDOM	411	16505	1.19	90.27	1.39	239
4	ENGLAND	383	16331	1.25	91.91	1.45	239
10	JAPAN	311	4897	0.76	83.60	0.74	46
6	GERMANY (FED REP GER)	298	7824	0.87	90.27	0.96	113
8	SOUTH KOREA	261	5146	0.84	86.21	0.86	35
9	ITALY	190	5010	0.96	93.68	1.20	151
7	NETHERLANDS	184	6675	1.13	97.28	1.40	82

In terms of citations, the United States ranks first with 36,220 citations (27.34 citations per paper, 88.60% of total citations). Mainland China ranks second with 21,724 citations (18.89 citations per paper, 85.48%), while the United Kingdom ranks third with 16,505 citations (40.16 citations per paper, 90.27%).

The CNCI value based on a single paper or a very small number of papers is insufficient to assess research impact; therefore, this study focuses on the top ten countries ranked by publication volume to evaluate their field-normalized citation impact. England has the highest CNCI value (1.45), followed by the Netherlands (1.40) and the United Kingdom (1.39). As for Collab-CNCI, England has the highest Collab-CNCI value (1.25), followed by the United Kingdom (1.19) and the Netherlands (1.13).

Using the number of patent citations as a measurement indicator, as shown in Table 1, among the top 10 countries by publication volume, the United States ranks first in patent citations in the HIFU field, with a total of 1,731 citations, followed by France with 405 citations. The United Kingdom ranks third, with 239 citations. These results suggest that the United States, France, and the United Kingdom place greater emphasis on technological innovation and the translational application of HIFU research outcomes.

#### 3.1.3 Research institution

**Figure 4:**
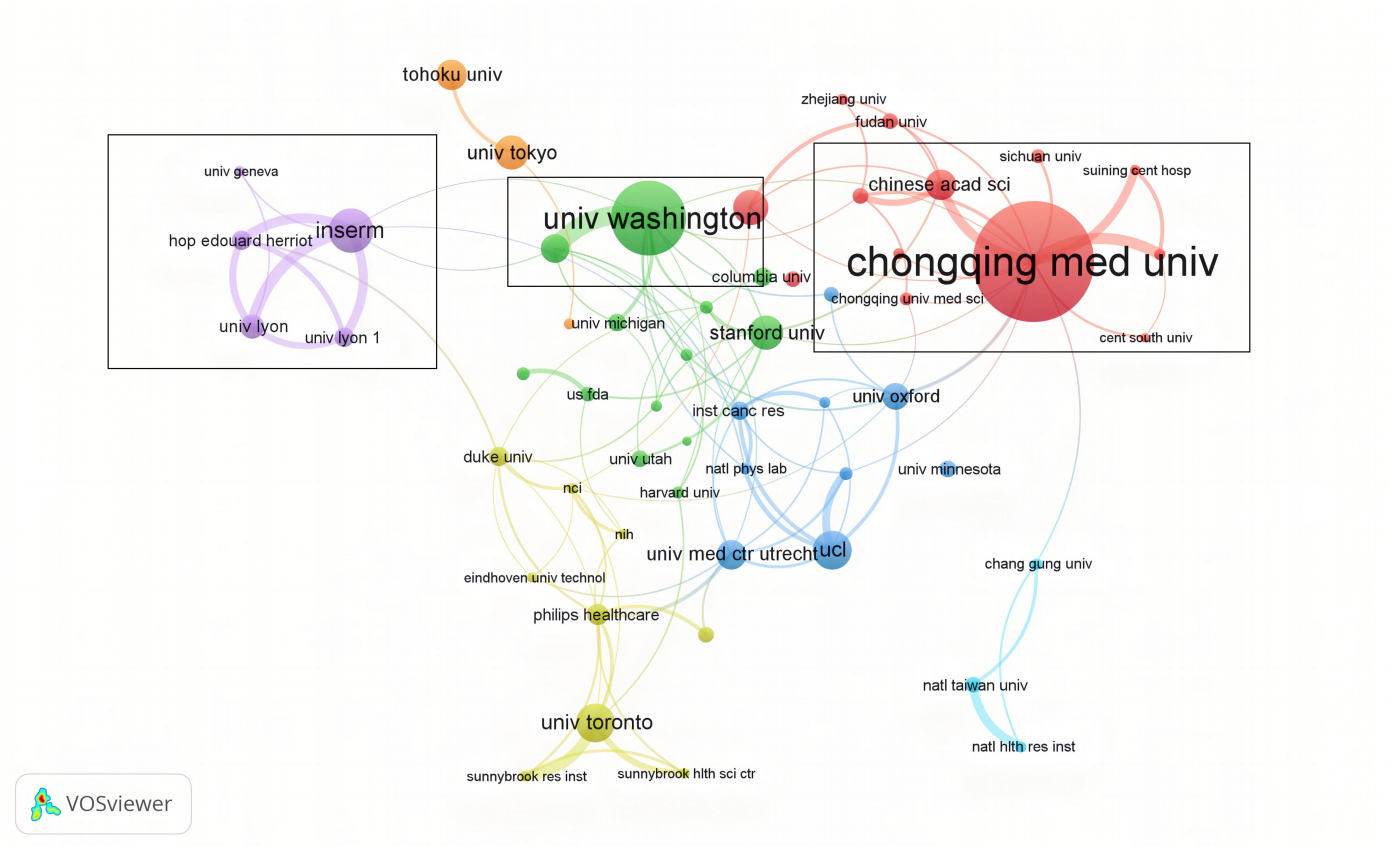
Illustrates the collaboration network among institutions engaged in HIFU research. Node size represents publication output, line thickness indicates collaboration strength, and colors denote different collaboration clusters rather than the average publication year. Chongqing Medical University and the University of Washington occupy central positions in the network.

In the field of HIFU, a total of 1,604 institutions contributed to publications between 1994 and 2024. The institutional distribution is illustrated in Figure 4, in which larger nodes represent institutions with higher publication volumes, including Chongqing Medical University in China, Inserm in France, and the University of Washington in the United States.

The collaboration network diagram reveals that Chongqing Medical University's partnerships are predominantly domestic, encompassing institutions such as Chongqing Haifu Hospital, the Chinese Academy of Sciences, Shanghai Jiao Tong University, and Zhejiang University. Internationally, it maintains close collaborative relationships with the University of Washington and Stanford University in the United States, as well as with the University of Oxford in the United Kingdom. The University of Washington has an even more extensive international collaboration network, encompassing Chongqing Medical University, the University of Tokyo in Japan, Inserm in France, Lomonosov Moscow State University, Cancer Research UK, and Philips in the Netherlands. In contrast, Inserm in France maintains a more limited network of international collaborations, partnering with Shanghai Jiao Tong University in China, the University of Washington, the University of Michigan, Lomonosov Moscow State University, and Cancer Research UK. The University of London's partner institutions are primarily based in the United Kingdom and include Imperial College London, Cancer Research UK, and the National Physical Laboratory. Its international partners include the University Medical Center Utrecht in the Netherlands and the University of Michigan in the United States.

As shown in Table 2, in terms of publication volume, Chongqing Medical University ranked first with 328 publications, followed by the Institut National de la Santé et de la Recherche Médicale (Inserm) with 249 publications. The University of Washington Seattle and the University of Washington tied for third place, with 203 publications each. In terms of citation count, Chongqing Medical University also ranked first, with a total of 10,732 citations, followed closely by the University of London with 8,571 citations. The University of Washington Seattle and the University of Washington tied for third place, with 7,716 citations each. Regarding the H-index, Chongqing Medical University ranked first with an H-index of 54, followed by the University of Washington Seattle and the University of Washington, both with an H-index of 49. The University of London ranked third, with an H-index of 47.

**Table 2:** Metrics for the top 10 research institutions by global publication volume: citations, share, h-index, and patent citations.

Rank	Name	Web of science documents	Times cited	% Docs cited	Category normalized citation impact	H-Index	Citations from patents
1	Chongqing Medical University	382	10732	89.79	1.08	54	92
2	Institut National de la Sante et de la Recherche Medicale (Inserm)	249	6675	82.33	0.97	44	241
3	University of Washington Seattle	203	7716	87.19	1.41	49	556
3	University of Washington	203	7716	87.19	1.41	49	556
4	University of London	198	8571	91.41	1.65	47	125
5	Center National de la Recherche Scientifique (CNRS)	126	3556	92.86	1.09	34	138
6	Philips	125	4634	94.40	1.29	36	147
7	University College London	115	3874	92.17	1.69	34	40
8	Universite Claude Bernard Lyon 1	107	2475	89.72	0.94	27	107
9	Universite Paris Cite	106	3453	92.45	1.26	36	60
10	Utrecht University	104	2692	96.15	1.20	30	40

Measured by patent citation count, the University of Washington leads by a wide margin, with 556 citations. INSERM ranks second with 241 citations, followed by Philips Corporation in third place with 147 patent citations. Another French research institution, CNRS, ranks fourth with 138 citations.

#### 3.1.4 Contributing authors

**Figure 5:**
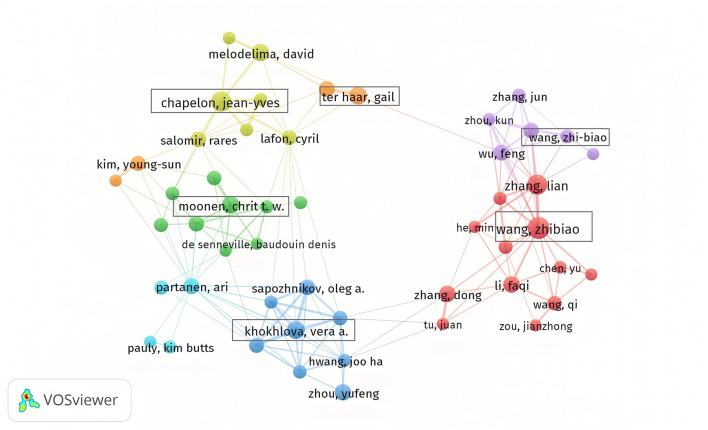
Co-authorship network of authors in HIFU research, generated using VOSviewer. Each node represents an author, and node size is proportional to publication output. Links indicate co-authorship relationships, with line thickness reflecting collaboration strength. Node colors denote distinct collaboration clusters rather than the average publication year. Prominent authors include Jean-Yves Chapelon, Christ T. W. Moonen, Vera A. Khokhlova, Gail ter Haar, Zhibiao Wang, and Lian Zhang, who occupy central positions within their respective collaborative groups.

In the field of HIFU, 14,408 researchers worldwide have published studies related to this topic. As shown in Figure 5, the larger nodes within each cluster represent highly productive authors, including Jean-Yves Chapelon from Inserm in France, Zhibiao Wang from Chongqing Medical University in China, and Vera A. Khokhlova from the University of Washington in the United States. The clustered network diagram indicates that the collaboration networks of highly productive authors primarily involve domestic researchers, whereas international collaborations are predominantly indirect.

As shown in Table 3, the top ten authors by publication volume are summarized. Jean-Yves Chapelon ranks first with 121 papers, accounting for 26.71% of the total, with an average of 31.20 citations per paper, a citation rate of 76.86%, and an H-index of 35. Zhibiao Wang ranks second with 88 papers, accounting for 19.43% of the total, with an average of 32.50 citations per paper, a citation rate of 93.18%, and an H-index of 30. Shin Yoshizawa ranks third with 84 papers, accounting for 18.54% of the total, with an average of 14.58 citations per paper, a citation rate of 82.14%, and an H-index of 22.

**Table 3:** Citation counts, affiliations, H-Index, and other metrics for the top ten researchers by publication volume.

Rank	Name	Web of science documents	Times cited	% Docs Cited	Affiliation	Category normalized citation impact	H-Index	Citations from patents
1	Chapelon, Jean-Yves	121	3775	76.86	Institut National de la Sante et de la Recherche Medicale (Inserm)	0.98	35	166
2	Wang, Zhibiao	88	2860	93.18	Chongqing Medical University	1.19	30	41
3	Yoshizawa, Shin	84	1225	82.14	Tohoku University	0.59	22	15
4	Khokhlova, Vera A	81	3192	88.89	University of Washington	1.62	27	89
5	HAAR, GR Ter	79	5137	91.14	University of London	1.80	30	104
6	Crum, Lawrence	73	3830	82.19	University of Washington Seattle	1.65	31	373
7	Melodelima, David	64	598	67.19	Institut National de la Sante et de la Recherche Medicale (Inserm)	0.46	15	5
8	Moonen, Chrit TW	60	2507	100.00	Universite de Bordeaux	1.37	30	119
9	Vaezy, S	57	2345	85.96	University of Washington Seattle	1.18	26	324
10	Gelet, A.	54	3409	96.30	University of Washington	1.82	30	80

**Figure 6:**
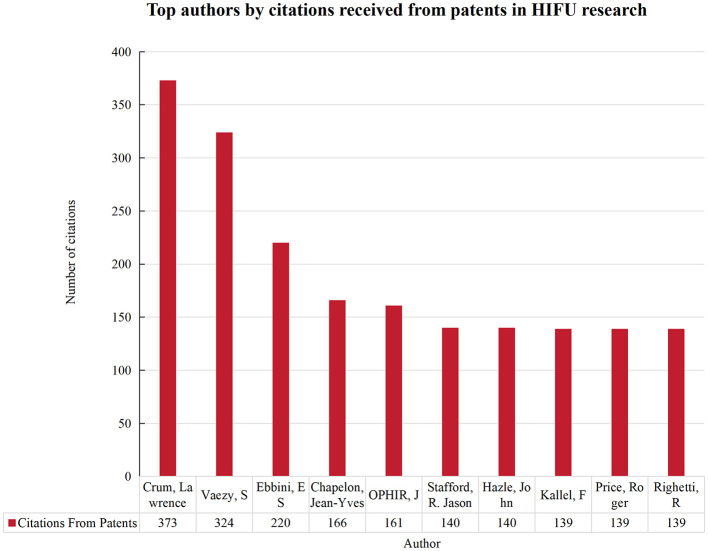
Top 10 authors by the number of patent citations received. The bar chart shows the total number of patent citations for each author, with Lawrence Crum receiving the highest number of citations (*n* = 373), followed by S. Vaezy (*n* = 324) and E. S. Ebbini (*n* = 220). These high patent citation counts reflect the strong translational and industrial impact of these authors' research, indicating their significant contributions to the development and commercialization of HIFU technology.

Figure 6 presents the ranking of authors based on patent citation count. Lawrence Crum from the University of Washington ranks first with 373 citations, followed by S. Vaezy from the same institution with 324 citations, E. S. Ebbini from the University of Minnesota Twin Cities with 220 citations, Jean-Yves Chapelon from INSERM with 166 citations, and J. Ophir from Tel Aviv University in Israel with 140 citations. When comparing the patent citation counts of the top 10 most prolific researchers, it is evident that although E. S. Ebbini and J. Ophir did not rank among the top 10 in terms of publication output, their patent citation counts placed them among the top 5 globally. This indicates that authors with high patent citation counts do not necessarily belong to the group of high-output researchers in their fields, and that there is no clear correlation between the volume of academic publications and the impact of patent technologies.

#### 3.1.5 Funding agencies

**Figure 7:**
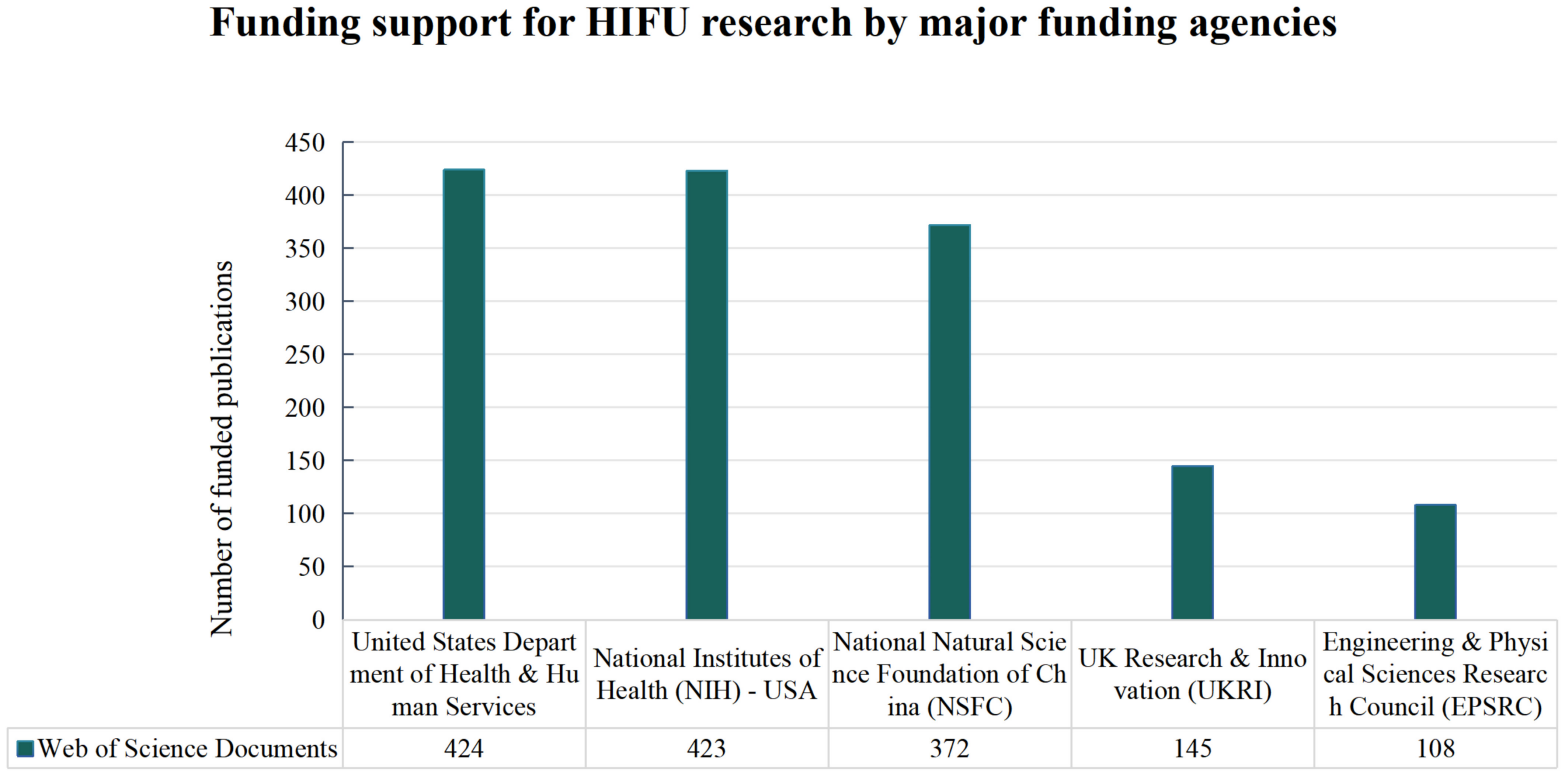
Major funding agencies supporting HIFU research. The bar chart displays the number of publications supported by leading funding bodies, as indexed in Web of Science. The U.S. Department of Health and Human Services (*n* = 424) and the National Institutes of Health (NIH, USA; *n* = 423) are the primary funders, followed by the National Natural Science Foundation of China (NSFC; *n* = 372). UK Research and Innovation (UKRI) and the Engineering and Physical Sciences Research Council (EPSRC) also represent significant sources of funding, reflecting the global landscape of financial support for HIFU research.

The extent to which countries prioritize HIFU research can be assessed by the number of funded HIFU-related projects. Globally, 288 institutions provide funding support for HIFU research. As shown in Figure 7, the top five funding agencies are the U.S. Department of Health and Human Services, with 424 projects; the U.S. National Institutes of Health, with 423 projects; the National Natural Science Foundation of China, with 372 projects; UK Research and Innovation, with 145 projects; and the Engineering and Physical Sciences Research Council in the United Kingdom, with 108 projects.

### 3.2 Evolutionary trends in research

#### 3.2.1 Keyword co-occurrence

**Figure 8:**
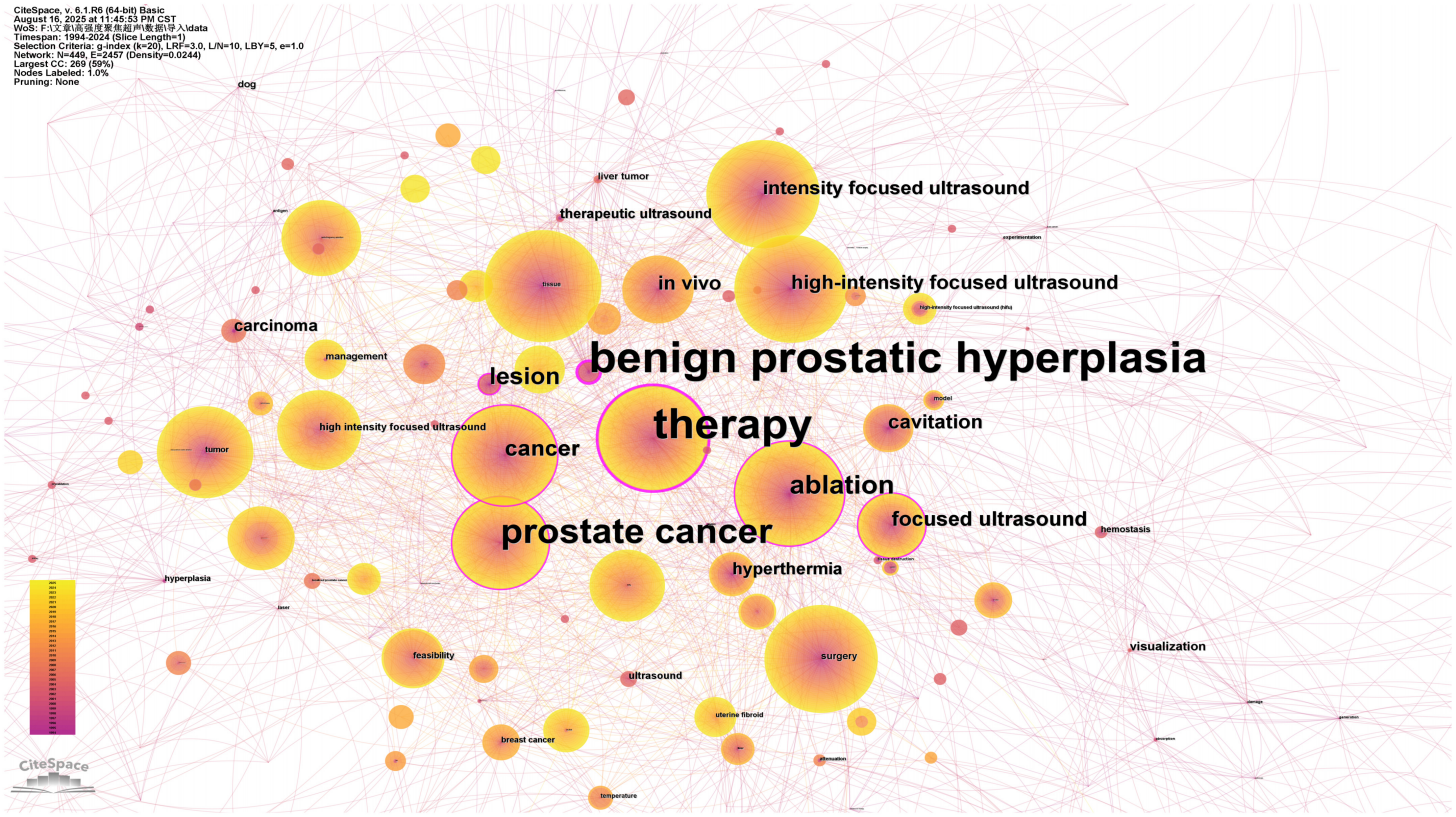
CiteSpace keyword co-occurrence network. Node size indicates keyword frequency, color represents temporal evolution, and links indicate co-occurrence. Major keywords include “benign prostatic hyperplasia,” “therapy,” “prostate cancer,” “ablation,” and “high-intensity focused ultrasound,” suggesting a focus on prostate disease treatment, ablation, and focused ultrasound applications.

Keyword co-occurrence analysis reveals research hotspots in the field of HIFU. Using CiteSpace 6.1.R6, a keyword co-occurrence map was generated, and the results were filtered according to betweenness centrality, as shown in Figure 8. Nodes with higher betweenness centrality are shown with thicker purple borders. The keyword with the highest betweenness centrality is “benign prostatic hyperplasia” at 0.22, followed by “therapy” at 0.21, “tissue ablation” at 0.19, “prostate cancer” at 0.18, and “enhancement” at 0.15. Other important keywords, including “lesion” at 0.12 and “cancer” at 0.11, function as mediators linking basic research to clinical applications, as shown in Table 4.

**Table 4:** Top 10 keywords by intermediary centrality.

Rank	Frequency	Intermediation centrality	Year	Keywords
1	18	0.22	1995	benign prostatic hyperplasia
2	675	0.21	1995	therapy
3	6	0.19	1997	tissue ablation
4	353	0.18	1998	prostate cancer
5	10	0.15	1999	enhancement
6	689	0.13	1996	ablation
7	15	0.12	1995	lesion
8	313	0.11	1996	cancer
9	675	0.10	1996	intensity focused ultrasound
10	672	0.10	1995	high-intensity focused ultrasound

#### 3.2.2 Keyword burst detection

Keyword bursts reflect rapidly evolving research hotspots in the field of HIFU from 1994 to 2024 and suggest potential future research trends. As shown in Table 5, 25 burst keywords were identified, with red bars indicating their burst periods. Chronologically, the earliest burst keyword was “carcinoma,” which had a burst period from 1995 to 2008 and a burst strength of 16.59. The second earliest was “benign prostatic hyperplasia” with a burst strength of 13.93 and a burst period from 1995 to 2004. The third earliest burst keyword was “hyperthermia,” with a burst strength of 12.35 and a burst period from 1995 to 2009. At the end of the timeline, keywords such as “management”, “safety”, “efficacy”, “uterine fibroid”, “outcome” and “focal therapy” showed the most recent bursts in prominence, with their periods of prominence concentrated between 2017 and 2024. Among them, “efficacy” (45.52), “management” (42.70), and “safety” (36.47) had the highest burst strengths. The relatively high burst strengths of “uterine fibroid” (31.30) and “outcome” (23.65). Notably, although “focal therapy” (12.65) had a relatively low burst strength, it emerged most recently, suggesting that this topic represents a frontier area in HIFU research and may become an important growth direction for future studies.

**Table 5:** Top 25 keyword burst table.

Top 25 keywords with the strongest citation bursts					
Keywords	Year	Strength	Begin	End	1994–2024
Carcinoma	1995	16.59	**1995**	**2008**	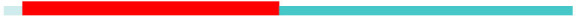
Benign prostatic hyperplasia	1995	13.93	**1995**	**2004**	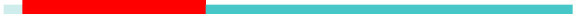
Hyperthermia	1995	12.35	**1995**	**2009**	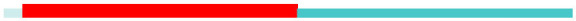
Lesion	1995	10.00	**1995**	**2005**	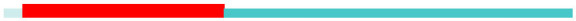
Tissue	1995	12.45	**2001**	**2006**	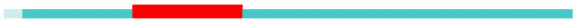
Radiation therapy	2003	19.17	**2003**	**2010**	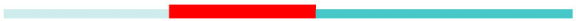
Experience	1997	43.34	**2005**	**2012**	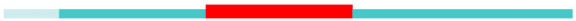
In vivo	1998	24.12	**2005**	**2015**	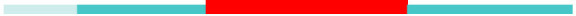
Radiotherapy	2006	13.33	**2006**	**2011**	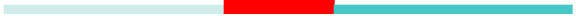
Temperature	1999	16.97	**2008**	**2011**	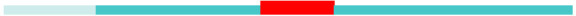
Feasibility	2003	23.50	**2009**	**2015**	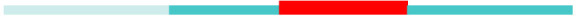
Breast cancer	1998	24.69	**2012**	**2016**	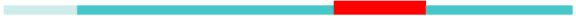
Cavitation	1995	16.87	**2012**	**2013**	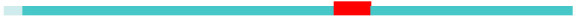
Drug delivery	2012	16.80	**2012**	**2014**	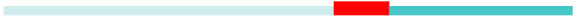
MRI	2003	17.38	**2013**	**2014**	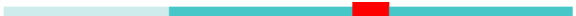
Hepatocellular carcinoma	1996	15.63	**2013**	**2015**	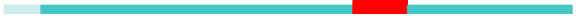
Liver	1996	11.55	**2013**	**2014**	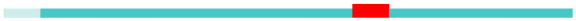
Radical prostatectomy	2005	13.82	**2015**	**2017**	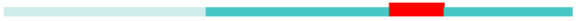
MR thermometry	2015	13.12	**2015**	**2016**	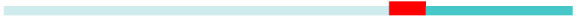
Management	1999	42.70	**2017**	**2024**	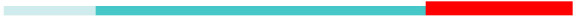
Safety	2017	36.47	**2017**	**2024**	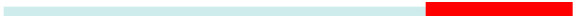
Efficacy	2019	45.52	**2019**	**2024**	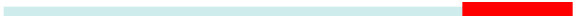
Uterine fibroid	2014	31.30	**2020**	**2024**	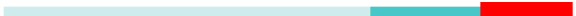
Outcome	2008	23.65	**2022**	**2024**	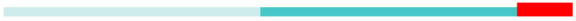
Focal therapy	2015	12.65	**2022**	**2024**	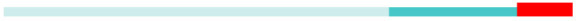

## 4 Discussion

### 4.1 Research trend analysis

Overall, HIFU research has progressed through three distinct phases. From 1994 to 2002, publication output was low and increased slowly, with an average of approximately 21 papers per year, indicating the initial stage of HIFU research. From 2003 to 2012, publications increased rapidly, with an average of approximately 150 papers per year, reflecting a period of accelerated growth. From 2013 to 2024, publication output continued to rise steadily despite fluctuations, with an average of approximately 264 papers per year, indicating a transition to a stable, high-output phase. Currently, however, publication growth has plateaued, likely because HIFU technology has matured within existing fields, increasing research complexity and creating a need to explore new research domains.

In its early stages, HIFU research consisted primarily of single-center studies and had not yet developed into a large-scale multicenter collaborative framework. Studies during this period focused mainly on animal models to explore the fundamental principles and technical feasibility of HIFU (ter Haar, 2021). Since 1994, the advantages of HIFU in minimally invasive and even non-invasive tumor treatment have continued to attract attention. Beginning in 1994, researchers in the United States published several papers exploring the feasibility of using HIFU to ablate hyperplastic prostatic tissue in animal models, thereby laying a foundation for understanding the technology's technical principles and establishing its experimental methodology (Bihrle et al., 1994). During the rapid growth phase of HIFU research, Zhibiao Wang's team at Chongqing Medical University in China developed the world's first HIFU-based tumor ablation system. This marked a major technological breakthrough in HIFU research, propelling the technology from laboratory research to clinical application, as evidenced by the surge in HIFU-related publications and the expansion of its clinical indications. During the maturation and standardization phase, publication output stabilized at a high level, and the HIFU technical system and its clinical applications became increasingly mature and standardized.

### 4.2 Country and institution analysis

In the field of HIFU, the United States has the largest publication volume, followed by China, and both countries far exceed all others in citation counts and overall output. France is also an active contributor. Although the United Kingdom produces fewer publications than these countries, its citation count, CNCI, and Collab-CNCI values rank among the top three globally, underscoring the high quality and disciplinary influence of its research.

The University of Washington is a leading institution in HIFU research and ranks second worldwide in H-index for HIFU-related publications, reflecting substantial institutional investment in the field. The University of Washington has demonstrated exceptional performance in international collaboration, indicating the strong international influence of its HIFU research. This facilitates the integration of resources, such as funding, data, and equipment, from different countries, enabling complementary strengths to be fully leveraged. The University of Washington's close collaboration with Philips in the Netherlands and Cancer Research UK demonstrates its strong emphasis on translating research findings into practical applications with real-world value. Its first-place ranking in patent citations further indicates a clear focus on research translation and applications with significant market potential. Research in the United States focuses on MRgFUS-based neurostimulation and functional brain disorders, localized cancer treatment and palliative pain management, focal therapy for prostate cancer, and blood–brain barrier opening and drug delivery to the brain (Meng et al., 2021; Eisenberg et al., 2021; Baal et al., 2021).

Chongqing Medical University is among the world's leading contributors to HIFU research and is the top institution in China in this field. The university was the birthplace of the world's first HIFU device, the “HIFU Knife,” which was developed in 1997 by Professor Zhibiao Wang's team. The team also pioneered the theory of the “biological focal zone” (Wang et al., 1997). Its research findings have been published in leading journals such as Ultrasonics Sonochemistry, forming the basis for the university's high-impact contributions. The collaborative network for HIFU research at Chongqing Medical University primarily involves partnerships with domestic universities, research institutes, and leading scientific institutions in Europe and the United States. In the future, the university can expand the scope of its international cooperation, strengthen exchanges with partners in Asia, Africa, and Latin America, and enhance collaboration with multinational medical companies to further increase its global influence. Among Western countries, France and the United Kingdom are the primary contributors to HIFU research. In particular, France's leading medical research institutions play a central role in advancing HIFU studies.

Research in France primarily focuses on prostate cancer treatment (Poissonnier et al., 2007; Thüroff et al., 2003) pericardial disease (Ninet et al., 2005), and varicose vein treatment (Delon-Martin et al., 1995). Publications from the United Kingdom are of high quality and are widely recognized internationally. HIFU research in the United Kingdom centers on focal therapy for prostate cancer, using HIFU's precise ablation capabilities to provide a function-preserving alternative to radical prostatectomy (Cordeiro et al., 2012).

### 4.3 Researcher contributions and collaboration patterns

The top ten global HIFU researchers display clear patterns of national distribution and institutional collaboration: four are from the United States, two are from France, and one each is from China, Japan, the United Kingdom, and Russia. The United States is represented by Vera A. Khokhlova's team, France by Jean-Yves Chapelon's team, and China by Zhibiao Wang's team.

Chapelon's institution integrates the full research continuum from basic science to clinical application, and his leading H-index reflects the prominent position of French researchers in HIFU research. Through collaborations with the Chinese Academy of Sciences and the University of Vermont, Wang's team has established an innovation network spanning original technological development, clinical validation, and international dissemination. Although Shin Yoshizawa's team at the University of Tokyo publishes extensively, its global citation impact remains modest, highlighting the tension between locally oriented technological applications and international outreach.

At the University of Washington, Vera A. Khokhlova, Lawrence Crum, Shahram Vaezy, and Arnaud Gelet form a major author cluster, demonstrating strong intra-institutional collaboration. Khokhlova's multinational publication record exemplifies transnational research activity, while Crum's global leadership in patent citations underscores the market-oriented value of the institution's HIFU research. In the United Kingdom, the HAAR and GT departments at University College London produce relatively fewer papers but achieve high H-index and CNCI values, reflecting a national tendency toward “fewer but higher-quality” publications.

It is worth noting that authors with a high number of patent citations, such as E. S. Ebbini and J. Ophir, are not necessarily high-output authors; this discrepancy may be due to differences in research focus and output objectives. Basic research emphasizes theoretical exploration and academic exchange, whereas patents focus on practical technological innovation and industrial application (Bignone and Lissoni, 2025). Furthermore, researchers may choose different forms of output based on their research orientation, leading to inconsistencies between the number of published papers and the impact of patents.

### 4.4 Funding agencies and research support

Funding agencies supporting HIFU research are concentrated in the United States, China, and the United Kingdom. In the United States, the U.S. Department of Health and Human Services (HHS) and the National Institutes of Health (NIH) serve as the primary funders. The National Natural Science Foundation of China ranks second globally, followed by UK Research and Innovation.

Adequate and sustained financial support is a key reason why the United States and China dominate global HIFU research. Thanks to substantial federal research funding, the United States has been able to sustain long-term exploration of fundamental mechanisms and pursue cutting-edge technological breakthroughs in this field; Ample funding has ensured a continuous influx of top talent and access to advanced experimental platforms, effectively driving the production of high-level theoretical achievements and original innovative research (Wu et al., 2021). Meanwhile, leveraging its robust national research investment capacity, China has relied on stable funding to vigorously advance research on HIFU clinical applications, large-scale clinical validation, and technology dissemination (Zhao et al., 2026). Ample research funding has effectively stimulated the enthusiasm of medical institutions and research teams, continuously expanding the scale of relevant research outputs and rapidly enhancing the field's overall academic influence. In contrast, although the United Kingdom has stable funding support, its overall scale of investment is relatively limited, making it difficult to establish a comprehensive advantage in terms of research volume and development momentum. These significant differences in funding scale and investment direction have directly widened the research gap among countries, ultimately establishing the dominant positions of the United States and China in the global HIFU research field.

### 4.5 Evolutionary pathways of HIFU research

Keywords with high betweenness centrality appeared relatively early, indicating that HIFU technology for solid tumor ablation gained broad recognition during its early stage of development. The timeline of keyword emergence further shows that HIFU research has evolved through three distinct phases.

First, the period from 1994 to 2004 represents the stage of foundational research and technological groundwork. Prominent keywords include carcinoma (16.59; 1995–2008), benign prostatic hyperplasia (13.93; 1995–2004), hyperthermia (12.35; 1995–2009), lesion (10.00; 1995–2005), tissue (12.45; 2001–2006), and radiation therapy (2003–2010). Early American studies confirmed the efficacy of HIFU in animal models and later expanded its application to tumors of the kidney, liver, breast, and brain (Illing et al., 2005; Wu et al., 2001; Watkin et al., 1997). Concurrently, researchers investigated the physical mechanisms underlying HIFU-induced tissue damage. Although HIFU is designed for precise ablation, heat may be conducted to surrounding tissues, causing damage to non-target areas. Therefore, HIFU treatment typically requires real-time monitoring using MRI (Schlesinger et al., 2013; Chen et al., 1997).

Second, from 2005 to 2016, HIFU technology underwent rapid development and clinical validation. Key themes during this period included “*in vivo*” (2005–2015), “radiotherapy” (2006–2011), “temperature” (2008–2011), “feasibility” (2009–2015), “cavitation” (2012–2013), “drug delivery” (2012–2014), “breast cancer” (2012–2016), and “hepatocellular carcinoma” (2013–2015), reflecting a focus on technological optimization and clinical trials. Research also explored the integration of HIFU with radiotherapy (Clarke and ter Haar, 1997), temperature monitoring (Bour et al., 2017; Hoogenboom et al., 2016), and cavitation effects (Kopechek et al., 2014; Peng et al., 2016; You et al., 2016), while advances in drug-delivery applications and MRI-guided HIFU precision ablation became major research priorities. Clinical studies during this phase primarily targeted breast, prostate, and liver cancers (Mougenot et al., 2009; Khokhlova et al., 2011; Jo and Yang, 2016; Kneepkens et al., 2016).

Third, the period from 2017 to 2024 represents the maturity and standardization phase. Key terms include “management” (2017–2024), “safety” (2017–2024), “efficacy” (2019–2024), “outcom” (2022–2024), “uterine fibroid” (2020–2024), and “focal therapy” (2022–2024). This phase emphasizes treatment management and safety and promotes the standardization of HIFU technology to minimize trauma through local ablative treatment. HIFU treatment for uterine fibroids offers advantages such as non-invasiveness, fertility preservation, and rapid recovery, making it a prioritized therapeutic approach in recent years (Dou et al., 2024; Delgado et al., 2025). “focal therapy” is an emerging concept that reflects researchers' growing interest in the precise and targeted application of HIFU. This shift toward organ-preserving, minimally invasive treatment underscores the evolution of HIFU from broad tissue ablation to personalized, site-specific therapeutic strategies (Bakavicius et al., 2022).

These developmental trajectories suggest that global HIFU research is now facing important opportunities. The expansion of clinical indications, the increasing demand for non-invasive therapies, and the integration of HIFU with imaging guidance, drug delivery, and precision medicine provide strong momentum for further development. At the same time, the field also faces significant challenges. As HIFU moves from technological exploration toward standardized clinical application, researchers are required to produce higher-quality evidence, conduct multicenter and large-scale clinical studies (Izadifar et al., 2020), improve methodological rigor, and address issues related to treatment reproducibility, long-term efficacy, and international standardization (Lewis et al., 2015). Therefore, although HIFU has shown considerable potential as a promising therapeutic modality, its future development will depend on the ability of researchers to balance technological innovation with clinical validation and standardized implementation.

### 4.6 Strengths and limitations

Using bibliometric methods, this study systematically characterizes the global research landscape, major research themes, and emerging trends in HIFU. It helps researchers and institutions better understand their positions within the global HIFU research landscape and identify potential opportunities for collaboration. Moreover, it provides researchers with a comprehensive overview of research progress, emerging frontiers, and future directions in HIFU, while highlighting critical scientific questions that warrant further investigation. Nevertheless, this study has several limitations. First, the use of a single database and the inclusion of only English-language publications may have introduced selection bias and may have led to the omission of some gray literature. Future studies should expand the range of databases and include multilingual literature to the greatest extent possible to reduce bias and improve the reliability and representativeness of the findings.

## 5 Conclusion

HIFU research has gone through three phases: a period of slow growth (1994–2002), a period of rapid growth (2003–2012), and a period of maturity and standardization (2013-present). Currently, the volume of publications has stabilized at a high level, and the field has entered a plateau phase. The global HIFU research landscape is characterized by a tripartite balance among the United States, China, and the United Kingdom and France: The United States leads in publication volume, citation counts, patent commercialization, and grant funding, driving technological innovation and the translation of research into practical applications; China, as a pioneer in this field, exemplified by Chongqing Medical University, has achieved high standards in both the quantity and quality of publications and is a core contributor to global HIFU research; the UK and France are renowned for their “small but excellent” high-quality research and strong disciplinary standardization influence, with the US and France leading globally in patent citations and demonstrating particularly outstanding capabilities in technology commercialization.

The United States, China, and the United Kingdom provide the most substantial research funding, fully demonstrating their forward-looking strategic positioning and robust capabilities in the field of biomedical engineering. The continuous optimization and innovation of HIFU technology are constantly expanding its range of indications, making it an important treatment option, particularly in the field of tumor ablation. However, the accumulation of large-scale, multicenter evidence-based data and systematic evaluations of safety and efficacy remain core areas of focus for future research.

It is worth noting that the dominance of English-language literature and the inclusion bias inherent in the Web of Science (WoS) database inevitably influence bibliometric results. The abundance of English-language publications from the United States and the United Kingdom significantly enhances their academic influence, whereas early research findings from China and France were often published in Chinese- or French-language journals, leading to a systematic underestimation of their academic contributions in global bibliometric analyses.

## Data Availability

The datasets presented in this study can be found in online repositories. The names of the repository/repositories and accession number(s) can be found in the article/supplementary material.
